# Drug-associated infections and infestations in older adults with tumor necrosis factor-alpha inhibitors: a real-world retrospective and pharmacovigilance study

**DOI:** 10.3389/fphar.2025.1533902

**Published:** 2025-04-30

**Authors:** Xihui Yu, Jiahong Zhong, Xuemei Zhuang, Zhuomiao Lin, Hongbo Fu, Yaofeng Zhang

**Affiliations:** ^1^ Department of Pharmacy, The Second Affiliated Hospital of Shantou University Medical College, Shantou, Guangdong, China; ^2^ Department of Clinical Pharmacy, Meizhou People’s Hospital (Huangtang Hospital), Meizhou, Guangdong, China; ^3^ Department of Pharmacy, Qingdao Central Hospital, University of Health and Rehabilitation Sciences, Qingdao, Shandong, China

**Keywords:** TNF-α inhibitor, immune-mediated inflammatory diseases, FAERS, older adults, infection, infestation

## Abstract

**Objective:**

One of the adverse events of greatest concern in patients receiving biologic therapies is the risk of infection, as infections are among the primary causes of premature mortality in this population, especially in the elderly. Because of the absence of head-to-head studies and limited duration and sample size of randomized controlled trials in the older adults, we analyzed the risk of infection associated with tumor necrosis factor (TNF) inhibitors by real-world adverse event analysis, seeking to identify medications with a reduced risk of infection and offering medication options for sensitive patients.

**Methods:**

A retrospective pharmacovigilance investigation was undertaken utilizing the FDA Adverse Event Reporting System (FAERS) database from the first quarter of 2010 to the fourth quarter of 2023. Drug-associated infections and infestations associated with TNF-α inhibitors (adalimumab, infliximab, etanercept, golimumab, and certolizumab pegol) were evaluated using a disproportionality analysis. The Reporting Odds Ratio (ROR) and Bayesian Confidence Propagation Neural Network (BCPNN) were utilized to detect AE signals.

**Results:**

A total case of 3,239,508 cases were included after removing duplicates. Among the protective signals, etanercept showed the lowest IC_025_ value in septic shock (IC_025_ = −3.23). Notably, golimumab showed the highest IC_025_ value in tuberculosis (IC_025_ = 2.44). The five TNF-α inhibitors have high signals in mycobacterial infectious disorders. In the stratification analysis, golimumab was associated with a highest risk of infections and infestations in ankylosing spondylitis patients (ROR = 3.07, 95%CI = 2.70–3.50; IC = 1.26, 95%CI = −0.42–2.92). Univariate and multivariate logistic regression analysis indicated that gender, weight and medicine may be influencing factors for the AEs of infections and infestations (p < 0.05).

**Conclusion:**

The research highlighted that the difference in the risk of infection in the elderly who used TNF-α inhibitors between various TNF-α inhibitors, adverse events and therapeutic indications, respectively. The use of TNF-α inhibitors increased the infection risk in older adults. Etanercept exhibited the lowest infection risk, whereas certolizumab pegol manifested the highest risk in infections and infestations. Doctors need to pay close attention to the appearance of mycobacterial infectious disorders in older adults treating with TNF-α inhibitors, which displayed the strongest signal.

## 1 Introduction

Immune-mediated inflammatory diseases (IMIDs), which are chronic ailments, share certain similar pathogenic processes and generate a considerable disease burden on millions of patients. Almost all IMIDs demonstrate higher levels of tumor necrosis factor (TNF), a versatile cytokine that interacts with multiple cell types and induces the production of numerous other inflammatory cytokines and chemokines (Monteleone et al., 2023). The development of biologic medications has contributed considerably to improving the clinical outcomes of IMIDs and as such these modalities have achieved first- or second-line positions in a wide range of treatment guidelines from different worldwide clinical associations. Tumor necrosis factor-alpha (TNF-α) inhibitors are effective treatments for IMIDs such rheumatoid arthritis, psoriatic arthritis, juvenile idiopathic arthritis, ulcerative colitis, psoriasis, Crohn’s disease and ankylosing spondylitis. At present, there are five Food and Drug Administration (FDA) approved biological TNF-α inhibitors, which are golimumab, etanercept, infliximab, adalimumab, and certolizumab pegol. While TNF-α inhibitors is clinically effective, there are concerns that it may increase the risk of infections due to its involvement in host defense.

Managing IMIDs in the elderly such as rheumatoid arthritis is challenging, since it is compounded by both comorbidities and polypharmacy (Galloway et al., 2011). Although the absolute number of immune cells does not diminish with age, studies show a functional drop in both innate and adaptive immunity, which includes cell-mediated and humoral immunity (El Chakhtoura et al., 2017). Infection is one of the most serious adverse effects (AEs) in patients receiving biologic therapy, since it is one of the leading causes of premature death in this population (Novella-Navarro and Balsa, 2022). This risk may be higher in the elderly due to the deterioration of the immune system with age. Multicenter prospective research conducted in a real-world setting showed that age at the commencement of biologic treatment is the most important risk factor for the occurrence of a first AE in rheumatoid arthritis, psoriatic arthritis and ankylosing spondylitis patients (Vela et al., 2020).

Because there are no head-to-head studies and the duration and sample size of randomized controlled trials in the elderly are restricted, direct information to compare the safety of existing TNF inhibitors is inadequate. As a result, we sought to examine the infection risk associated with TNF inhibitors using a real-world retrospective and pharmacovigilance investigation in order to identify medications with a reduced risk of infection and make medication recommendations for susceptible patients.

## 2 Materials and methods

### 2.1 Data source and collection

We conducted a retrospective pharmacovigilance study on AEs of five TNF-α inhibitors (etanercept, adalimumab, infliximab, golimumab and certolizumab pegol) based on the FDA Adverse Event Reporting System (FAERS) database, which is a publicly available database of safety reports submitted by consumers, pharmacists, and pharmaceutical companies around the world since 2004. It is an invaluable resource for monitoring drug safety by aggregating global adverse event reports submitted mainly by healthcare professionals, patients, and pharmaceutical companies, allowing for comprehensive real-world analyses (Zhong et al., 2024; Li et al., 2025). AEs were collected from the first quarter of 2010 to the fourth quarter of 2023.

### 2.2 Data processing

The FAERS data were retrieved from the Quarterly Data Extract Files, which are publicly accessible at https://fis.fda.gov/extensions/FPD-QDE-FAERS/FPD-QDE-FAERS.html. We download the FAERS data and clean the data by RStudio following the recommendations from the FDA. Both generic and brand names were used to identify TNF-α inhibitor-associated infections and infestations records: etanercept (ETANERCEPT, ETANERCEPT SZZS, ENBREL, TNFR FC, ERELZI), adalimumab (ADALIMUMAB, ADALIMUMAB ATTO, ADALIMUMAB ADBM, HUMIRA, HUMIRA 40 MG 0 8 ML PEN, AMJEVITA, CYLTEZO), infliximab (REMICADE, INFLECTRA, RENFLEXIS, INFLIXIMAB, INFLIXIMAB DYYB, INFLIXIMAB ABDA), golimumab (GOLIMUMAB, SIMPONI) and certolizumab pegol (CERTOLIZUMAB PEGOL, CIMZIA, CDP870, CDP 870). Only the reports of these TNF-α inhibitors with role code as the primary suspected drug were chosen for analysis.

Preferred terms (PTs) from the Medical Dictionary for Regulatory Activities (MedDRA) should be utilized for standardized encoding when referring to AEs names in the reports. All PTs grouped into a broader category of infections and infestations known as system-organ classes (SOC) were included in the study.

For reports with the same CASEID, we retain the one with the largest FDA_DT value. For reports where both CASEID and FDA_DT are the same, we retain the one with the largest PRIMARYID value. The cleaned and standardized data was compiled into a final dataset, which is ready for analysis. This dataset included only those reports where these five TNF-α inhibitors was listed as the primary suspected drug (PS), aligning with our study’s focus. All AEs reports for these five TNF-α inhibitors were analyzed at the high-level group term (HLGT) and PT levels.

### 2.3 Statistical analysis

In the data mining process, we employed the Reporting Odds Ratio (ROR) and information component (IC) to lessen the bias of false-positive findings induced by one approach. The equations and criteria for the two algorithms are described in Supplementary Table S1. Both Frequentist (the reporting odds ratio [ROR]) and Bayesian (the information component [IC]) methods in the disproportionality analysis were employed to explore the relationship between TNF-α inhibitors and AEs of infections and infestations. For ROR, it was defined a signal if the lower limit of the 95% confidence interval (ROR_025_) more than one, with at least three cases. For IC, the lower limit of the 95% confidence interval of IC (IC_025_) exceeding zero was considered as a potential drug-associated infections and infestations adverse arrhythmic reaction signal. AEs signals that satisfied both algorithm criteria were considered significant signals. Data were analyzed using Microsoft Excel 2021 and R 4.3.0.

## 3 Results

### 3.1 General characteristics

**FIGURE 1:**
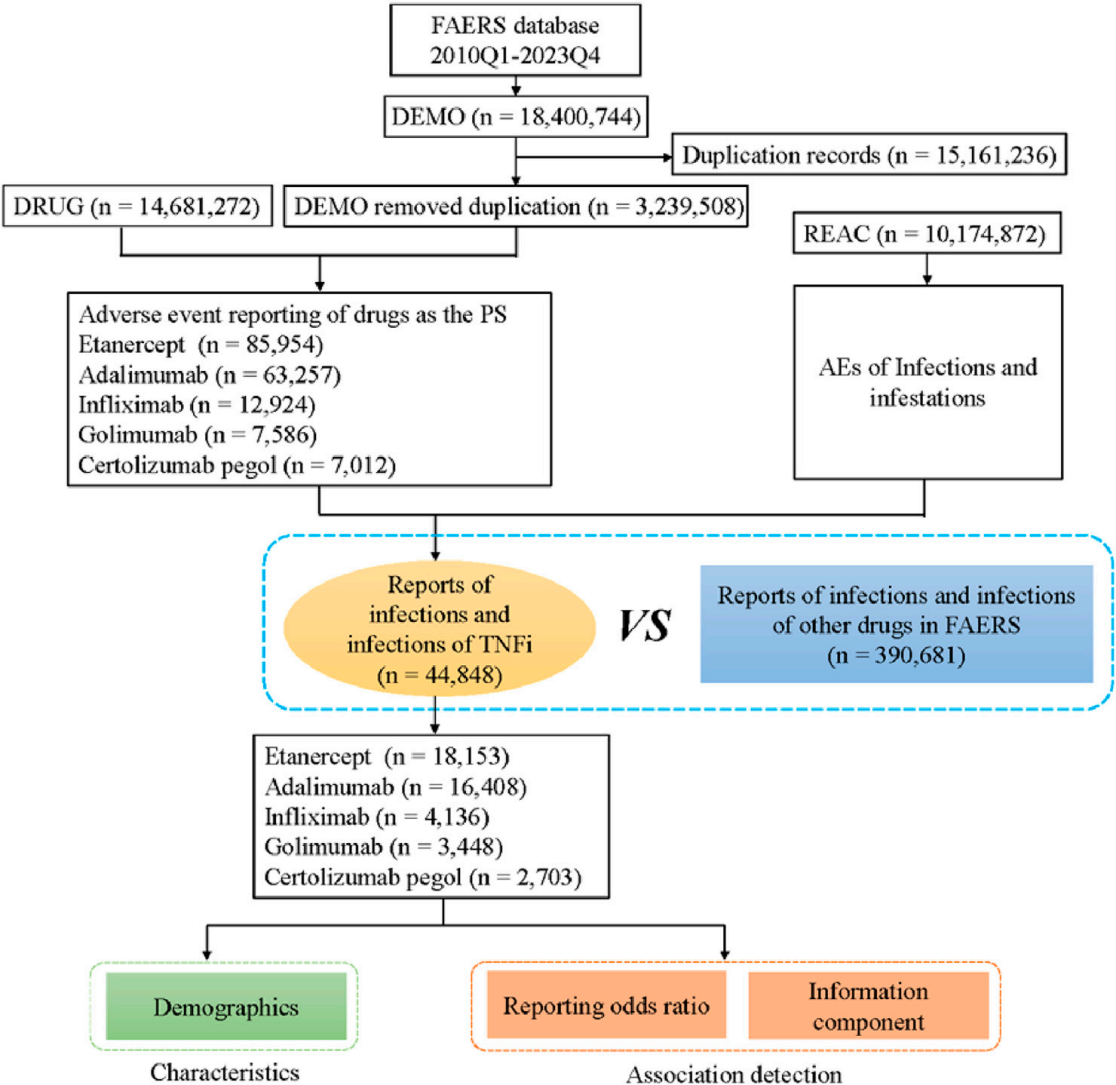
Flow diagram of the study (DEMO, demographic and administrative information; DRUG, drug Information; REAC, preferred terminology for adverse drug reactions; PS, primary suspect drug).

From the first quarter of 2010 to the fourth quarter of 2023, 18,400,744 reports related to five TNF-α inhibitors were obtained from the FAERS database. A total case of 3,239,508 cases were remained after removing duplicates and 44,848 AEs were associated with five TNF-α inhibitors (Figure 1). The numbers of AE reports of infections and infestations for etanercept, adalimumab, infliximab, golimumab and certolizumab pegol were 18,153 (40.48%), 16,408 (36.59%), 4,136 (9.22%), 3,448 (7.69%) and 2,703 (6.03%), respectively.

Clinical characteristics of AEs for different TNF-α inhibitors are shown in Table 1. In terms of gender, more female patients (73.06%) reported drug-associated infections and infestations in older adults. The vast majority of reports focused on the age group from 65 to 74 years (70.17%), followed by the age group of 75–84 years (26.26%). The median age (interquartile range) of older adults was 71 (67–76) years, and the highest median age was observed in the golimumab group. The United States (55.23%) was the main reporting country for these AEs. The vast majority of reports were provided by consumer (45.70%). Excluding unknown outcome, hospitalization (30.87%) gets the most reports and the time to onset of infections and infestations in >360 days group (11.60%) occupy a large proportion.

**TABLE 1:** Demographic data on reports of infections and infestations related to drug in treatment with TNF-α inhibitors in older adults in the FAERS database.

Characteristic	Cases (percentage)					
	All TNF-α inhibitors	Etanercept	Adalimumab	Infliximab	Golimumab	Certolizumab pegol
Total Cases	44,848	18,153	16,408	4,136	3,448	2,703
Gender						
Female	32,765 (73.06%)	14,176 (78.09%)	11,592 (70.65%)	2,427 (58.68%)	2,549 (73.93%)	2,021 (74.77%)
Male	11,844 (26.41%)	3,902 (21.50%)	4,702 (28.66%)	1,682 (40.67%)	884 (25.64%)	674 (24.94%)
Unknown or missing	239 (0.53%)	75 (0.41%)	114 (0.69%)	27 (0.65%)	15 (0.44%)	8 (0.30%)
Age						
65–74	31,470 (70.17%)	12,820 (70.62%)	11,628 (70.87%)	2,867 (69.32%)	2,241 (64.99%)	1,914 (70.81%)
75–84	11,779 (26.26%)	4,713 (25.96%)	4,212 (25.67%)	1,117 (27.01%)	1,044 (30.28%)	693 (25.64%)
>84	1,599 (3.57%)	620 (3.42%)	568 (3.46%)	152 (3.68%)	163 (4.73%)	96 (3.55%)
Median (IQR)	71 (67–76)	71 (67–76)	71 (67–76)	71 (68–76)	72 (68–77)	71 (67–75)
Reporting conutry						
United States	24,771 (55.23%)	13,901 (76.58%)	8,379 (51.07%)	811 (19.61%)	507 (14.70%)	1,173 (43.40%)
Canada	5,015 (11.18%)	725 (3.99%)	707 (4.31%)	2,196 (53.09%)	972 (28.19%)	415 (15.35%)
Other countries	15,062 (33.58%)	3,527 (19.43%)	7,322 (44.62%)	1,129 (27.30%)	1,969 (57.11%)	1,115 (41.25%)
Outcome						
Death	2,680 (5.98%)	714 (3.93%)	1,154 (7.03%)	446 (10.78%)	208 (6.03%)	158 (5.85%)
Life-threatening	602 (1.34%)	156 (0.86%)	225 (1.37%)	109 (2.64%)	53 (1.54%)	59 (2.18%)
Disability	134 (0.30%)	44 (0.24%)	59 (0.36%)	14 (0.34%)	8 (0.23%)	9 (0.33%)
Hospitalization	13,843 (30.87%)	3,880 (21.37%)	6,108 (37.23%)	1,533 (37.06%)	1,317 (38.20%)	1,005 (37.18%)
Congenital anomaly	3 (0.01%)	0 (0.00%)	1 (0.01%)	1 (0.02%)	0 (0.00%)	1 (0.04%)
Required intervention to prevent permanent impairment/damage	8 (0.02%)	4 (0.02%)	3 (0.02%)	1 (0.02%)	0 (0.00%)	0 (0.00%)
Other serious (important medical event)	11,449 (25.53%)	4,593 (25.30%)	2,582 (15.74%)	1,710 (41.34%)	1,426 (41.36%)	1,138 (42.10%)
Unknown	16,129 (35.96%)	8,762 (48.27%)	6,276 (38.25%)	322 (7.79%)	436 (12.65%)	333 (12.32%)
Reported person						
Healthcare profession	3,855 (8.60%)	621 (3.42%)	1,008 (6.14%)	1,412 (34.14%)	632 (18.33%)	182 (6.73%)
Physician	11,897 (26.53%)	7,430 (40.93%)	2,174 (13.25%)	853 (20.62%)	761 (22.07%)	679 (25.12%)
Pharmacist	878 (1.96%)	337 (1.86%)	192 (1.17%)	72 (1.74%)	182 (5.28%)	95 (3.51%)
Other health-professional	4,786 (10.67%)	2,456 (13.53%)	744 (4.53%)	780 (18.86%)	466 (13.52%)	340 (12.58%)
Consumer	20,496 (45.70%)	6,001 (33.06%)	10,824 (65.97%)	994 (24.03%)	1,320 (38.28%)	1,357 (50.20%)
Lawyer	4 (0.01%)	1 (0.01%)	1 (0.01%)	2 (0.05%)	0 (0.00%)	0 (0.00%)
Unknown or missing	2,932 (6.54%)	1,307 (7.20%)	1,465 (8.93%)	23 (0.56%)	87 (2.52%)	50 (1.85%)
The time to onset (days)						
0–7	382 (0.85%)	94 (0.52%)	143 (0.87%)	34 (0.82%)	53 (1.54%)	58 (2.15%)
8–30	917 (2.04%)	226 (1.24%)	380 (2.32%)	73 (1.76%)	130 (3.77%)	108 (4.00%)
31–90	1,392 (3.10%)	347 (1.91%)	534 (3.25%)	96 (2.32%)	200 (5.80%)	215 (7.95%)
91–180	1,048 (2.34%)	277 (1.53%)	428 (2.61%)	58 (1.40%)	149 (4.32%)	136 (5.03%)
181–360	1,192 (2.66%)	334 (1.84%)	472 (2.88%)	63 (1.52%)	161 (4.67%)	162 (5.99%)
>360	5,204 (11.60%)	1,838 (10.13%)	1,778 (10.84%)	474 (11.46%)	613 (17.78%)	501 (18.53%)
Unknown or missing	34,713 (77.40%)	15,037 (82.83%)	12,673 (77.24%)	3,338 (80.71%)	2,142 (62.12%)	1,523 (56.34%)
Reporting years						
2010–2011	2,144 (4.78%)	849 (4.68%)	753 (4.59%)	359 (8.68%)	81 (2.35%)	102 (3.77%)
2012–2013	5,970 (13.31%)	3,580 (19.72%)	1,427 (8.70%)	574 (13.88%)	235 (6.82%)	154 (5.70%)
2014–2015	7,393 (16.48%)	3,986 (21.96%)	2,330 (14.20%)	444 (10.74%)	345 (10.01%)	288 (10.65%)
2016–2017	8,433 (18.80%)	3,858 (21.25%)	3,120 (19.02%)	400 (9.67%)	574 (16.65%)	481 (17.80%)
2018–2019	6,646 (14.82%)	2,706 (14.91%)	2,499 (15.23%)	319 (7.71%)	506 (14.68%)	616 (22.79%)
2020–2021	5,977 (13.33%)	1,358 (7.48%)	2,596 (15.82%)	766 (18.52%)	749 (21.72%)	508 (18.79%)
2022–2023	8,285 (18.47%)	1,816 (10.00%)	3,683 (22.45%)	1,274 (30.80%)	958 (27.78%)	554 (20.50%)

### 3.2 Signal detection

**FIGURE 2:**
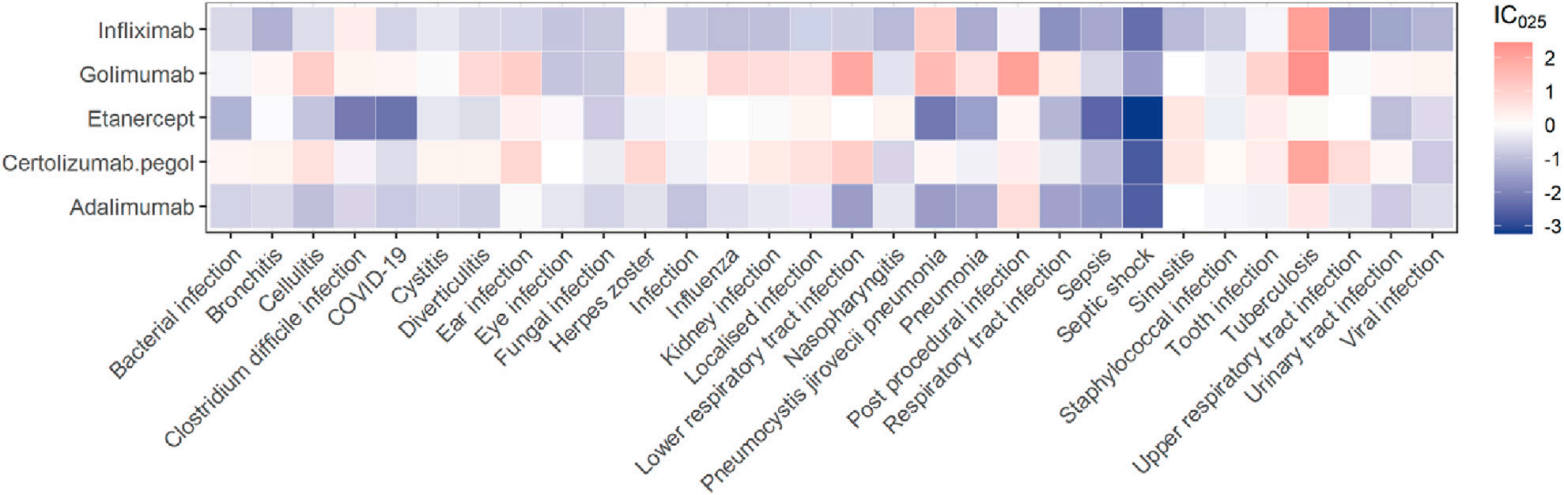
A heatmap of IC_025_ showing the associations between TNF-α inhibitors and the top 30 PTs of infections and infestations ranked by report numbers.

The association spectrum of the five TNF-α inhibitors in infections and infestations is shown in Figure 2. The red presents that the lower limit of the 95% confidence interval of IC (IC_025_) is greater than zero, which is considered a significant signal. The top 30 signal strength of TNF-α inhibitors at the preferred term (PT) level is shown at Supplementary Table S2. It was obviously observed that tuberculosis has a high level of signal intensity in the five TNF-α inhibitors. Golimumab and certolizumab pegol had higher level of signal intensity than others in infection and infestation-related AEs, respectively. Among the protective signals, etanercept showed the lowest IC_025_ value in septic shock (IC_025_ = −3.23). Notably, golimumab showed the highest IC_025_ value in tuberculosis (IC_025_ = 2.44). We marked significant signals in red font at Supplementary Table S2. Golimumab and certolizumab pegol exhibited a greater number of significant signals than others. Tuberculous infection was present in the five TNF-α inhibitors. Eye infection was only observed in certolizumab pegol (ROR_025_ = 1.95, IC_025_ = 0.02). Urinary tract infections (such as cystitis), abdominal and gastrointestinal infections (such as diverticulitis), upper respiratory tract infections and lower respiratory tract infections (such as bronchitis) should be given more attention when golimumab or certolizumab pegol was used.

**FIGURE 3:**
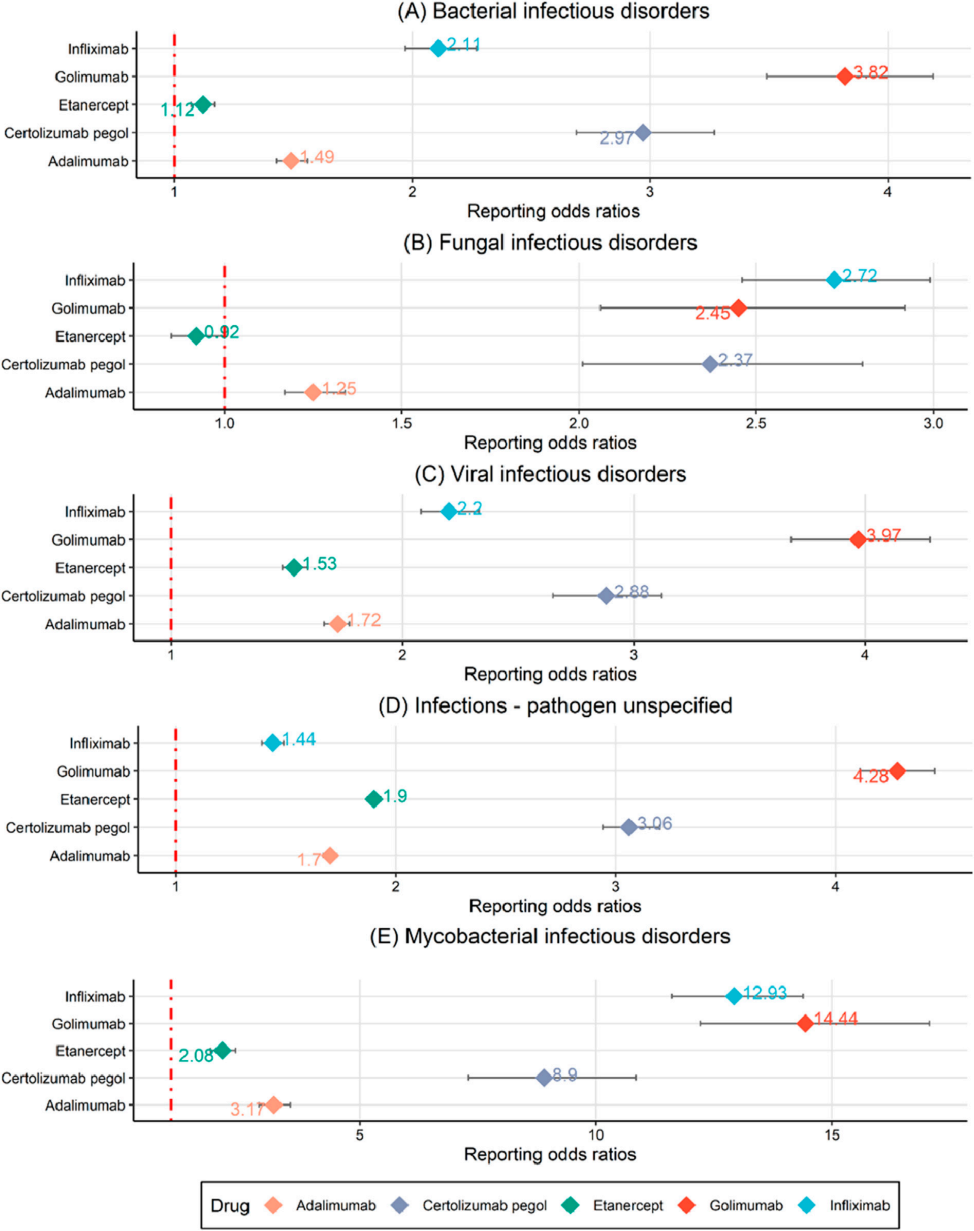
A disproportionality analysis through reporting odds ratios at the HLGT level. **(A)** Bacterial infectious disorders. **(B)** Fungal infectious disorders. **(C)** Viral infectious disorders. **(D)** Infections - pathogen unspecified. **(E)** Mycobacterial infectious disorders.

In addition to performing a disproportionate analysis at the PT level, HLGTs were selected to group PTs into broader categories and further assess the association between TNF-α inhibitors and events of infections and infestations in Figure 3 and Supplementary Figure S1. There are twelve classes of HLGTs in infections and infestations (Supplementary Figure S2). Since there are too few cases in some classes, we finally observed the association between TNF-α inhibitors and the five classes of HLGTs (bacterial infectious disorders, fungal infectious disorders, viral infectious disorders, infections - pathogen unspecified and mycobacterial infectious disorders). Among the five classes of HLGTs, only etanercept had negative signals for fungal infectious disorders and others had positive signals. It's worth noting that the five TNF-α inhibitors have high ROR and IC value in mycobacterial infectious disorders.

After limiting the patients to the same indications, the association between the five TNF-α inhibitors and drug-associated infections and infestations showed in Table 2. In the stratification analysis, golimumab was associated with a highest risk of infections and infestations in ankylosing spondylitis patients (ROR = 3.07, 95%CI = 2.70–3.50; IC = 1.26, 95%CI = −0.42–2.92). We found that golimumab showed positive signs in rheumatoid arthritis, psoriatic arthropathy, psoriasis, Crohn disease, ankylosing spondylitis and ulcerative colitis patients at ROR_025_ level (ROR_025_ > 1). Certolizumab pegol also showed positive signs in these indications without ulcerative colitis.

**TABLE 2:** Reporting of infections and infestations related to drug in treatment with TNF-α inhibitors with various indications.

TNF-α inhibitor	Indication	Case	ROR (95% CI)	IC (95% CI)
Etanercept	Rheumatoid Arthritis	15,656	0.80 (0.79, 0.82)	−0.22 (−1.89, 1.44)
	Psoriatic Arthropathy	2,557	0.93 (0.89, 0.97)	−0.07 (−1.74, 1.59)
	Psoriasis	1993	0.78 (0.74, 0.82)	−0.28 (−1.94, 1.39)
	Crohn’s disease	6	0.57 (0.25, 1.29)	−0.77 (−2.38, 1.00)
	Ankylosing Spondylitis	510	0.78 (0.70, 0.86)	−0.27 (−1.93, 1.40)
	Ulcerative Colitis	8	1.55 (0.74, 3.24)	0.56 (−1.23, 2.17)
Adalimumab	Rheumatoid Arthritis	11,542	0.90 (0.88, 0.92)	−0.11 (−1.78, 1.55)
	Psoriatic Arthropathy	2,387	0.91 (0.87, 0.96)	−0.09 (−1.76, 1.57)
	Psoriasis	3,190	0.95 (0.91, 0.99)	−0.06 (−1.72, 1.61)
	Crohn’s Disease	3,491	0.87 (0.83, 0.91)	−0.11 (−1.78, 1.56)
	Ankylosing Spondylitis	786	0.93 (0.86, 1.02)	−0.06 (−1.73, 1.60)
	Ulcerative Colitis	1,425	0.99 (0.93, 1.05)	−0.01 (−1.68, 1.65)
Infliximab	Rheumatoid Arthritis	2,173	1.23 (1.18, 1.29)	0.26 (−1.41, 1.92)
	Psoriatic Arthropathy	377	1.04 (0.93, 1.16)	0.05 (−1.62, 1.71)
	Psoriasis	201	1.34 (1.16, 1.56)	0.38 (−1.30, 2.04)
	Crohn’s Disease	1,675	0.99 (0.93, 1.04)	−0.02 (−1.68, 1.65)
	Ankylosing Spondylitis	242	1.07 (0.93, 1.23)	0.08 (−1.59, 1.75)
	Ulcerative Colitis	983	1.00 (0.93, 1.08)	0.00 (−1.67, 1.67)
Golimumab	Rheumatoid Arthritis	3,118	2.50 (2.40, 2.60)	1.07 (−0.59, 2.74)
	Psoriatic Arthropathy	392	2.78 (2.48, 3.11)	1.22 (−0.45, 2.89)
	Psoriasis	26	1.66 (1.09, 2.51)	0.64 (−1.08, 2.28)
	Crohn’s Disease	35	2.27 (1.57, 3.26)	1.03 (−0.69, 2.66)
	Ankylosing Spondylitis	338	3.07 (2.70, 3.50)	1.26 (−0.42, 2.92)
	Ulcerative Colitis	147	2.01 (1.68, 2.40)	0.88 (−0.80, 2.54)
Certolizumab pegol	Rheumatoid Arthritis	2,767	1.76 (1.69, 1.84)	0.68 (−0.99, 2.35)
	Psoriatic Arthropathy	366	1.55 (1.38, 1.73)	0.54 (−1.13, 2.21)
	Psoriasis	104	1.43 (1.17, 1.76)	0.46 (−1.22, 2.12)
	Crohn’s Disease	275	2.01 (1.76, 2.28)	0.87 (−0.80, 2.53)
	Ankylosing Spondylitis	149	1.53 (1.28, 1.83)	0.52 (−1.16, 2.18)
	Ulcerative Colitis	7	0.77 (0.36, 1.65)	−0.35 (−2.01, 1.36)

**FIGURE 4:**
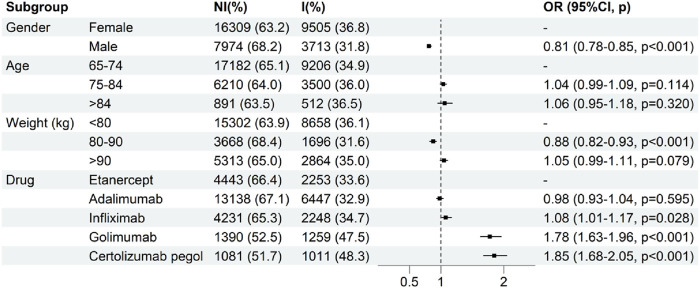
Multivariate logistic regression analysis of the odds ratio for adverse events of drug-associated infections and infestations (NI, Not infections and infestations; I, Infections and infestations).

Logistic regression analysis between patient gender, age, weight, different TNF-α inhibitors and the AEs of infections and infestations was presented in Figure 4 and Supplementary Figure S3. Univariate and multivariate logistic regression analysis indicated that gender, weight and drug may be influencing factors for the AEs of infections and infestations (p < 0.05). Our study found that the risk of AEs related to infections and infestations increased by 1.78 times and 1.85 times when older adults use golimumab (OR = 1.78, p < 0.001) and certolizumab pegol (OR = 1.85, p < 0.001) rather than etanercept, respectively. The risk of drug-associated infections and infestations for male older adults (OR = 0.81, p < 0.001) was 0.81 times that of the female older adults. The risk of AEs related to infections and infestations for older adults in 80–90 kg (OR = 0.88, p < 0.001) was 0.88 times that of older adults in <80 kg.

## 4 Discussion

Immune system is significantly changed with age, contributing to the increased risk of infections and autoimmune illness in older adults (Bueno et al., 2014). Patients with rheumatoid arthritis, psoriatic arthritis and ankylosing spondylitis often require life-long therapy. Therefore, long-term safety is a crucial concern for both healthcare professionals and patients when considering treatment. The established infection may result in extended hospitalization or possibly be life-threatening. Drug-associated infections and infestations may impair the quality of living of the elderly and pose a major burden on the family. TNF-α inhibitors are commonly used to treat numerous chronic autoimmune diseases such as rheumatoid arthritis, Crohn’s disease, systemic lupus erythematosus, ulcerative colitis, psoriasis and psoriatic arthropathy. However, patients taking TNF-α inhibitors may have increased invasive fungal infection risk, which can be life threatening and lead to need withdrawal of TNF-α inhibitor therapy (Vallabhaneni and Chiller, 2016). Infections are the most prevalent class of AEs reported in phase 3 randomized trials of golimumab (Husni et al., 2022). In order to evaluate the infection risk of TNF-α inhibitors from a large sample viewpoint, we conducted a real-world retrospective and pharmacovigilance analysis utilizing FARES database. To our knowledge, this is the first comprehensive pharmacovigilance research to assess the connection between TNF-α inhibitors and AEs of infections and infestations in older adults. We assessed of the infection risk among the elderly with five different TNF-α inhibitors. The results demonstrated all TNF-α inhibitors increased the risk of any infection, with certolizumab pegol displaying the highest risk. The production of infection may be related to the suppression of TNF-α, which can diminish cell death and depress proper immune responses during microbial infections (van Loo and Bertrand, 2023). Etanercept reduced the risk of fungal infectious diseases. Two meta-analyses imply that etanercept is a potentially safer alternative for infections (Michaud et al., 2014; Jiang et al., 2024), where we reached to the same conclusion.

According to the descriptive analysis, females (73.06%) made up the majority of TNF-α inhibitor-related infections and infestations. Our multivariate logistic regression analysis revealed that elderly women were more vulnerable to infection when treated with TNF-α inhibitors. In the various age categories, 65–74 years (70.17%) accounted for the greater amount. These traits might be related to the epidemiological features of chronic autoimmune diseases. Globally, females have a greater incidence rate and prevalence of rheumatoid arthritis. The global age-standardized incidence rate is higher in females and rises with age, peaking in 2019 in the 70–74 and 75–79 age groups for females and males, respectively (Shi et al., 2023). Except for unknown or missing cases, the duration to start was greater in the >360 days group, indicating a higher risk beyond 360 days. It indicates that the use of TNF-α inhibitors require careful long-term follow-up.

In addition, our study revealed a differential association between TNF-α inhibitors and AEs from infections and infestations. We found that etanercept showed more of the lowest signal values in ROR and IC among five TNF-α inhibitors through disproportionality analysis in Figure 3. This may be due to the fact that etanercept only binds soluble TNF well in most cases and other species in a reversible manner with a high off-rate, whereas other TNF-α inhibitors bind free TNF, transmembrane TNF and receptor-bound TNF irreversibly (Keane, 2005). A meta-analysis showed that etanercept had a decreased risk of tuberculosis and general infections compared with mono-antibodies (Liao et al., 2017). Thus, we chose etanercept as a control to compare the risk of infection and infestations between TNF-α different inhibitors. As shown in Figure 4, golimumab and certolizumab pegol exhibited higher risk of infections and infestations than that of etanercept, which had significant relationship, respectively. A systematic review and network meta-analysis also showed that certolizumab pegol displayed the highest infection risk among five different TNF inhibitors (Jiang et al., 2024).

Among the different infection types, the five TNF-α inhibitors had the strongest signal in mycobacterial infectious disorders and the weakest signal in fungal infectious disorders (Figure 3). In Figure 2, it is evident that golimumab exhibits a high heat map signal in tuberculosis, followed by infliximab, certolizumab pegol, and adalimumab, with only etanercept showing a signal value close to 0. The use of TNF-α inhibitors enhances tuberculosis susceptibility and incidence by 1.6–25.1 times (Kim et al., 2023). This could be explained by the effect of its blockade in the T-helper 1 (Th1) response, especially interferon-γ-mediated. It plays a critical role in host defense against tuberculosis and breakdown of tuberculosis granuloma which is thought to act as a source of tuberculosis dissemination (Ehlers, 2003). Furthermore, TNF-α inhibitors reduce the apoptosis of macrophages infected with tuberculosis and hinder immune cell migration, reducing tuberculosis clearance and raising risk (Yasui, 2014). To reduce the danger of reactivating latent tuberculosis infection, clinicians should test for both main and latent tuberculosis before starting TNF-α treatment. Etanercept is suggested above other TNF-α inhibitors to minimize the risk of tuberculosis infection in individuals with latent tuberculosis infection reactivation or previously treated tuberculosis (Godfrey and Friedman, 2019).

Although fungal infections disorders showed relatively low signal values at the HLGT level, the high morbidity and mortality associated with them cannot be ignored, especially invasive fungal disease (Candel et al., 2020). TNF-α inhibitors may limit the Th1-type cell response, preventing the formation of a granulomatous response and promoting fungal dispersion. As a general rule, immunosuppressive medication should be discontinued until the infection is entirely controlled (Baddley et al., 2018).

The difference in infection risk for TNF-α inhibitors to be mostly explained by their different molecular structures, leading to differences in pharmacokinetics and mechanisms of action. Etanercept is structurally similar to the receptor and does not subsequently activate the complement, neither causes antibody-dependent cytotoxicity nor induces T-cell apoptosis (Candel et al., 2020). Owing to its rapid dissociation rate, etanercept sheds about 50% of soluble TNF and 90% of transmembrane TNF only 10 min after binding. Infliximab, adalimumab and golimumab are full IgG1 monoclonal antibodies against human TNF-a (Mitoma et al., 2018). With a strong binding affinity for both monomeric and trimeric TNF, infliximab is a chimeric monoclonal antibody composed of 75% human and 25% murine components. It seldom ever releases soluble or transmembrane TNF once it is coupled to them, and it forms highly stable complexes with both types of TNF. Golimumab is a human monoclonal antibody that targets TNF, while adalimumab is a completely human monoclonal IgG1 antibody. Compared to etanercept, infliximab, adalimumab, and golimumab exhibited higher affinity, strength, and stability for TNF binding (Smith, 2010). Certolizumab pegol is the only PEGylated TNF-α inhibitor with a higher binding affinity of TNF compared to other TNF-α inhibitors, which may enhance its pharmacokinetics and prolong half-life (Pasut, 2014).

Rates of AEs in patients treated with TNF inhibitors may vary across therapeutic indications due to demographic variations, such as disease-inherent risks and frequency of comorbidities (Burmester et al., 2013). Patients with rheumatoid arthritis who use TNF-α inhibitors are more likely to get significant infections than those with psoriatic arthritis or ankylosing spondylitis (Christensen et al., 2022). We can also draw the same conclusion in Table 2 where most cases are in rheumatoid arthritis for each TNF-α inhibitor.

Our study relies on real-world pharmacovigilance data and gave in-depth insights on AEs of infections and infestations in older adults following the usage of TNF-α inhibitors. An notable advantage over prior research is that this study acquired a significant number of population reports in the presence of the elderly. Nevertheless, this retrospective pharmacovigilance study exists some limitations. Firstly, the data are spontaneously provided by consumers, physicians and others in the FAERS database, with different data quality, correctness, and completeness. Secondly, it is difficult to draw a causal conclusion since it relies on observational reports, which is a frequent flaw in all pharmacovigilance research. Thirdly, the data predominantly originate from American (55.23%) and Canadian (11.18%) populations, with comparatively few reported data from other groups.

## 5 Conclusion

Based on real-world pharmacovigilance data, the current study systematically evaluated the association between TNF-α inhibitors and AEs of infections and infestations, providing substantial contributions to clinical practice and medication safety research. This study identified that the use of TNF-α inhibitors increased the infection risk in older adults. Etanercept had a lowest risk while certolizumab pegol displayed the highest risk in infections and infestations. Among the different infection types, we should focus on mycobacterial infectious disorders with the strongest signal, while fungal infectious disorders with the lowest signal cannot be ignored. It is worth noting that patients with rheumatoid arthritis are more likely to develop infections undergoing treatment with TNF-α inhibitors. The findings of this study provide the difference in infection risk in the elderly who used TNF-α inhibitors between different TNF-α inhibitors, AEs and therapeutic indications, respectively, which provides medication suggestions for susceptible patients.

## Data Availability

Publicly available datasets were analyzed in this study. This data can be found here: https://fis.fda.gov/extensions/FPD-QDE-FAERS/FPD-QDE-FAERS.html.

## Abbreviations

TNF, tumor necrosis factor; IMIDs, Immune-mediated inflammatory diseases; AEs, adverse events; FAERS, FDA Adverse Event Reporting System; PTs, preferred terms; SOC, system organ class; HLGT, high-level group term; ROR, reporting odds ratio; PRR, proportional reporting ratio; BCPNN, Bayesian Confidence Propagation Neural Network; MedDRA, Medical Dictionary for Regulatory Activities.
