# Supplementary material for: Drug-associated infections and infestations in older adults with tumor necrosis factor-alpha inhibitors: a real-world retrospective and pharmacovigilance study
Source: Front Pharmacol. 2025 Apr 30;16:1533902. doi: 10.3389/fphar.2025.1533902 (PMC12075941; doi:10.3389/fphar.2025.1533902)
Supplement: Supplementary file 1 [file Table1.docx]

Supplementary Material

# Supplementary Tables

**Supplementary Table 1.** Calculation Formula and Standard of Signal Detection

| Algorithm | Calculation Formula | Criterion |
| --- | --- | --- |
| ROR | $\mathrm{ROR}=\frac{a/c}{b/d}=\frac{ad}{bc}$ | a ≥ 3 |
|  | $95\%CI=e^{lnROR\pm1.96\sqrt{\frac{1}{a}+\frac{1}{b}+\frac{1}{c}+\frac{1}{d}}}$ | The lower limit of 95%CI (ROR_025_) > 1 |
| BCPNN | IC = ${log}_{2}\frac{a(a+b+c+d)}{(a+b)(a+c)}$ |  |
|  | $E\left( IC \right)={log}_{2}\frac{(a+\gamma_{11})(N+\alpha)(N+\beta)}{(N+\gamma)(a+b+\alpha_{1})(a+c+\beta_{1})}$  $V\left( IC \right)=\frac{1}{{(ln2)}^{2}}[\frac{N-a+\gamma-\gamma_{11}}{(a+\gamma_{11})(N+1+\gamma)}+\frac{N-a-b+\alpha-\alpha_{1}}{(a+b+\alpha_{1})(N+1+\alpha)}+\frac{N-a-c+\beta-\beta_{1}}{(a+c+\beta_{1})(N+1+\beta)}]$  $\gamma=\gamma_{11}\frac{(N+\alpha)(N+\beta)}{(a+b+\alpha_{1})(a+c+\beta_{1})}$  $95\%CI=E(IC)\pm1.96\sqrt{V(IC)}$  Where α=α_1_+α_2_, β=β_1_+β_2_, N=a+b+c+d, and the value of α_1_, α_2_, β_1_, β_2_ and γ_11_ were defined as 1. | The lower limit of 95%CI (IC_025_) > 0 |

Abbreviations:

a, number of reports containing both the target drug and target adverse drug reaction; b, number of reports containing other adverse drug reaction of the target drug; c, number of reports containing the target adverse drug reaction of other drugs; d, number of reports containing other drugs and other adverse drug reactions; 95%CI, 95% confidence interval; IC, information component; IC_025_, the lower limit of 95% CI, of the IC; ROR, reporting odds ratio.

**Supplementary Table 2.** The top 30 signal strengths of TNF-α inhibitor ranked by report numbers at the PTs level in the FAERS database

| PTs | All TNF-α inhibitors | | | Etanercept | | | Adalimumab | | | Infliximab | | | Golimumab | | | Certolizumab pegol | | |
| --- | --- | --- | --- | --- | --- | --- | --- | --- | --- | --- | --- | --- | --- | --- | --- | --- | --- | --- |
|  | Case | ROR_025_ | IC_025_ | Case | ROR_025_ | IC_025_ | Case | ROR_025_ | IC_025_ | Case | ROR_025_ | IC_025_ | Case | ROR_025_ | IC_025_ | Case | ROR_025_ | IC_025_ |
| Pneumonia | 6840 | 1.36 | -1.22 | 2348 | 1.08 | -1.5 | 2514 | 1.18 | -1.38 | 627 | 1.17 | -1.33 | 823 | 4.72 | 0.61 | 528 | 2.58 | -0.2 |
| Nasopharyngitis | 4437 | 3.18 | -0.13 | 2397 | 3.88 | 0.24 | 1567 | 2.45 | -0.35 | 229 | 1.33 | -1.07 | 124 | 2.02 | -0.4 | 120 | 1.73 | -0.63 |
| Urinary tract infection | 4243 | 1.81 | -0.83 | 1563 | 1.52 | -1.01 | 1793 | 1.78 | -0.8 | 280 | 1.06 | -1.42 | 289 | 3.25 | 0.18 | 318 | 3.18 | 0.14 |
| Infection | 2883 | 2.38 | -0.48 | 1509 | 2.85 | -0.15 | 872 | 1.59 | -0.93 | 213 | 1.49 | -0.9 | 158 | 3.19 | 0.22 | 131 | 2.3 | -0.23 |
| Sinusitis | 2774 | 4.27 | 0.23 | 1472 | 4.94 | 0.57 | 979 | 3.15 | 0 | 112 | 1.26 | -1.07 | 80 | 2.56 | 0 | 131 | 3.92 | 0.54 |
| COVID-19 | 2694 | 1.41 | -1.15 | 521 | 0.6 | -2.26 | 1408 | 1.75 | -0.81 | 376 | 1.83 | -0.66 | 231 | 3.23 | 0.19 | 158 | 1.89 | -0.53 |
| Influenza | 2608 | 2.92 | -0.22 | 1268 | 3.17 | 0 | 883 | 2.16 | -0.5 | 149 | 1.34 | -1.01 | 181 | 4.91 | 0.82 | 127 | 2.95 | 0.13 |
| Bronchitis | 2278 | 2.71 | -0.31 | 1150 | 3.08 | -0.04 | 775 | 2.02 | -0.59 | 119 | 1.13 | -1.23 | 106 | 2.94 | 0.15 | 128 | 3.20 | 0.25 |
| Herpes zoster | 1944 | 3.07 | -0.15 | 795 | 2.73 | -0.18 | 652 | 2.22 | -0.45 | 245 | 3.27 | 0.2 | 100 | 3.62 | 0.46 | 152 | 5.08 | 0.89 |
| Sepsis | 1356 | 0.85 | -1.81 | 356 | 0.51 | -2.48 | 626 | 0.94 | -1.64 | 182 | 1.06 | -1.37 | 104 | 1.71 | -0.62 | 88 | 1.25 | -1.04 |
| Cellulitis | 1214 | 2.01 | -0.66 | 423 | 1.54 | -0.92 | 402 | 1.48 | -0.98 | 133 | 1.88 | -0.52 | 139 | 5.85 | 1.1 | 117 | 4.28 | 0.67 |
| Cystitis | 1107 | 2.4 | -0.43 | 479 | 2.31 | -0.38 | 392 | 1.87 | -0.65 | 116 | 2.09 | -0.35 | 52 | 2.50 | 0.04 | 68 | 3.01 | 0.25 |
| Lower respiratory tract infection | 1075 | 2.77 | -0.26 | 544 | 3.12 | 0.02 | 181 | 0.94 | -1.55 | 78 | 1.57 | -0.71 | 168 | 10.86 | 1.95 | 104 | 5.64 | 1.09 |
| Upper respiratory tract infection | 1006 | 2.93 | -0.19 | 482 | 3.09 | 0.02 | 377 | 2.38 | -0.32 | 31 | 0.61 | -1.87 | 40 | 2.42 | 0.05 | 76 | 4.47 | 0.8 |
| Localised infection | 965 | 3.39 | -0.01 | 473 | 3.62 | 0.23 | 324 | 2.39 | -0.3 | 59 | 1.52 | -0.7 | 50 | 3.71 | 0.61 | 59 | 3.96 | 0.67 |
| Diverticulitis | 837 | 2.25 | -0.5 | 340 | 2.02 | -0.54 | 293 | 1.73 | -0.74 | 78 | 1.7 | -0.59 | 72 | 4.58 | 0.85 | 54 | 2.92 | 0.26 |
| Staphylococcal infection | 717 | 2.79 | -0.23 | 296 | 2.49 | -0.24 | 311 | 2.66 | -0.16 | 49 | 1.42 | -0.76 | 25 | 1.9 | -0.18 | 36 | 2.59 | 0.17 |
| Kidney infection | 572 | 2.84 | -0.2 | 255 | 2.74 | -0.1 | 213 | 2.26 | -0.34 | 33 | 1.15 | -0.97 | 37 | 3.88 | 0.74 | 34 | 3.11 | 0.45 |
| Viral infection | 522 | 2.1 | -0.57 | 219 | 1.93 | -0.56 | 221 | 1.98 | -0.53 | 34 | 1.01 | -1.17 | 31 | 2.66 | 0.25 | 17 | 1.14 | -0.8 |
| Ear infection | 506 | 3.74 | 0.14 | 231 | 3.59 | 0.27 | 181 | 2.75 | -0.06 | 29 | 1.41 | -0.65 | 32 | 4.68 | 1.05 | 33 | 4.3 | 0.91 |
| Clostridium difficile infection | 500 | 1.72 | -0.82 | 84 | 0.58 | -2.13 | 235 | 1.85 | -0.63 | 116 | 3.58 | 0.41 | 35 | 2.69 | 0.23 | 30 | 1.99 | -0.17 |
| Fungal infection | 485 | 1.86 | -0.72 | 192 | 1.62 | -0.8 | 209 | 1.8 | -0.66 | 44 | 1.32 | -0.85 | 15 | 1.07 | -0.85 | 25 | 1.77 | -0.28 |
| Tooth infection | 460 | 3.75 | 0.15 | 224 | 3.88 | 0.37 | 151 | 2.49 | -0.18 | 37 | 2.09 | -0.15 | 27 | 4.24 | 0.95 | 21 | 2.77 | 0.41 |
| Tuberculosis | 446 | 7.21 | 0.86 | 111 | 3.01 | 0.11 | 145 | 4.18 | 0.52 | 107 | 12.37 | 2.13 | 45 | 13.22 | 2.44 | 38 | 9.58 | 2.02 |
| Respiratory tract infection | 382 | 1.31 | -1.16 | 163 | 1.26 | -1.13 | 128 | 0.97 | -1.46 | 25 | 0.63 | -1.76 | 39 | 3.16 | 0.44 | 27 | 1.81 | -0.27 |
| Post procedural infection | 378 | 5.24 | 0.54 | 120 | 3.10 | 0.14 | 184 | 5.22 | 0.78 | 23 | 1.9 | -0.17 | 38 | 10.21 | 2.11 | 13 | 2.43 | 0.39 |
| Eye infection | 318 | 2.56 | -0.29 | 152 | 2.66 | -0.09 | 123 | 2.11 | -0.38 | 21 | 1.13 | -0.87 | 7 | 0.81 | -0.9 | 15 | 1.95 | 0.02 |
| Pneumocystis jirovecii pneumonia | 307 | 1.73 | -0.78 | 49 | 0.53 | -2.17 | 74 | 0.86 | -1.55 | 112 | 5.84 | 1.10 | 50 | 6.86 | 1.49 | 22 | 2.3 | 0.13 |
| Bacterial infection | 303 | 1.72 | -0.79 | 96 | 1.15 | -1.19 | 134 | 1.7 | -0.69 | 34 | 1.49 | -0.61 | 16 | 1.77 | -0.14 | 23 | 2.44 | 0.2 |
| Septic shock | 278 | 0.41 | -2.75 | 83 | 0.27 | -3.23 | 125 | 0.43 | -2.62 | 37 | 0.46 | -2.32 | 22 | 0.73 | -1.52 | 11 | 0.27 | -2.7 |

Notes: Red font indicates important signals (ROR_025_ > 1, IC_025_ > 0).

# Supplementary Figures

**
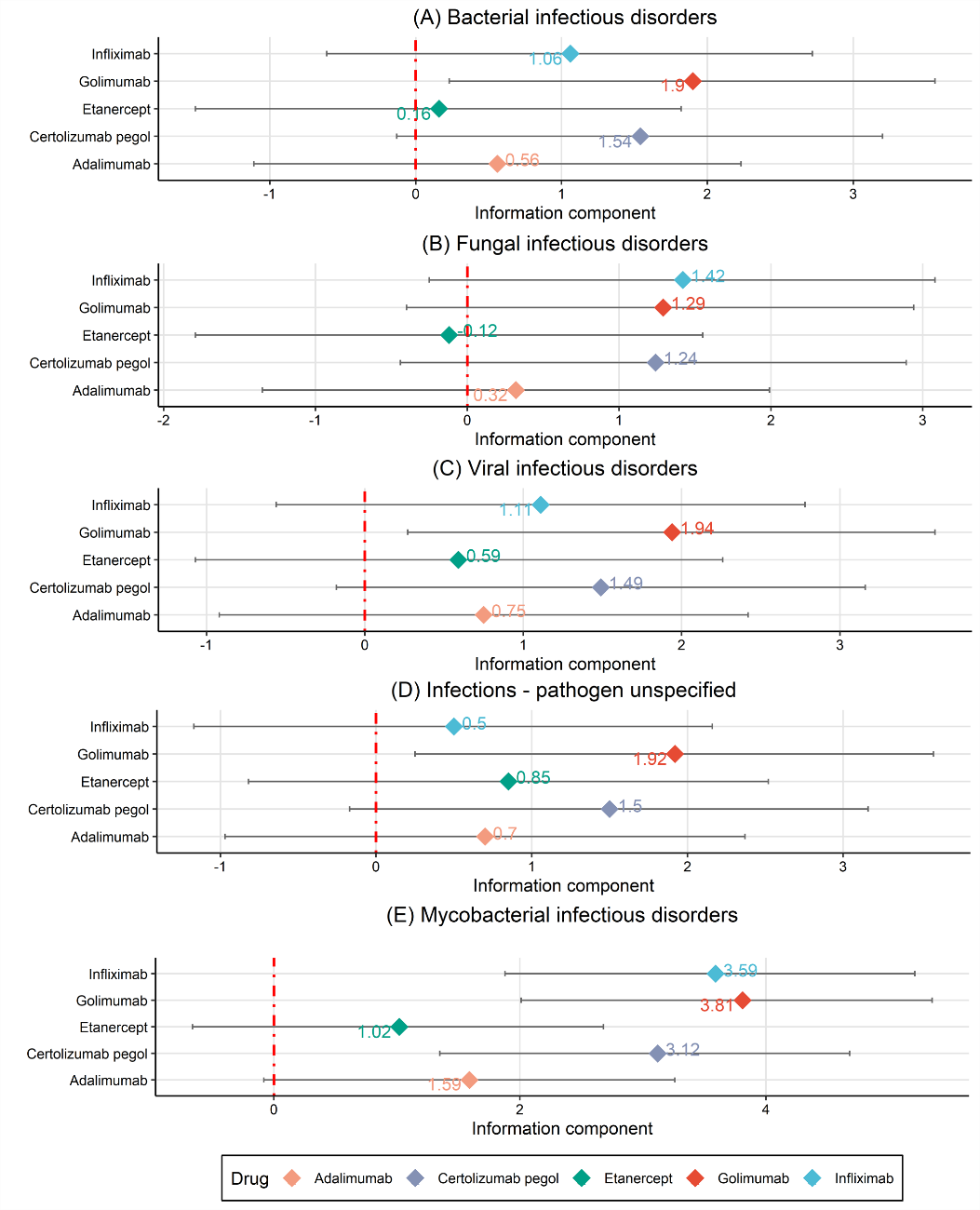
**

**Supplementary Figure 1.** A disproportionality analysis through information component at the HLGT level


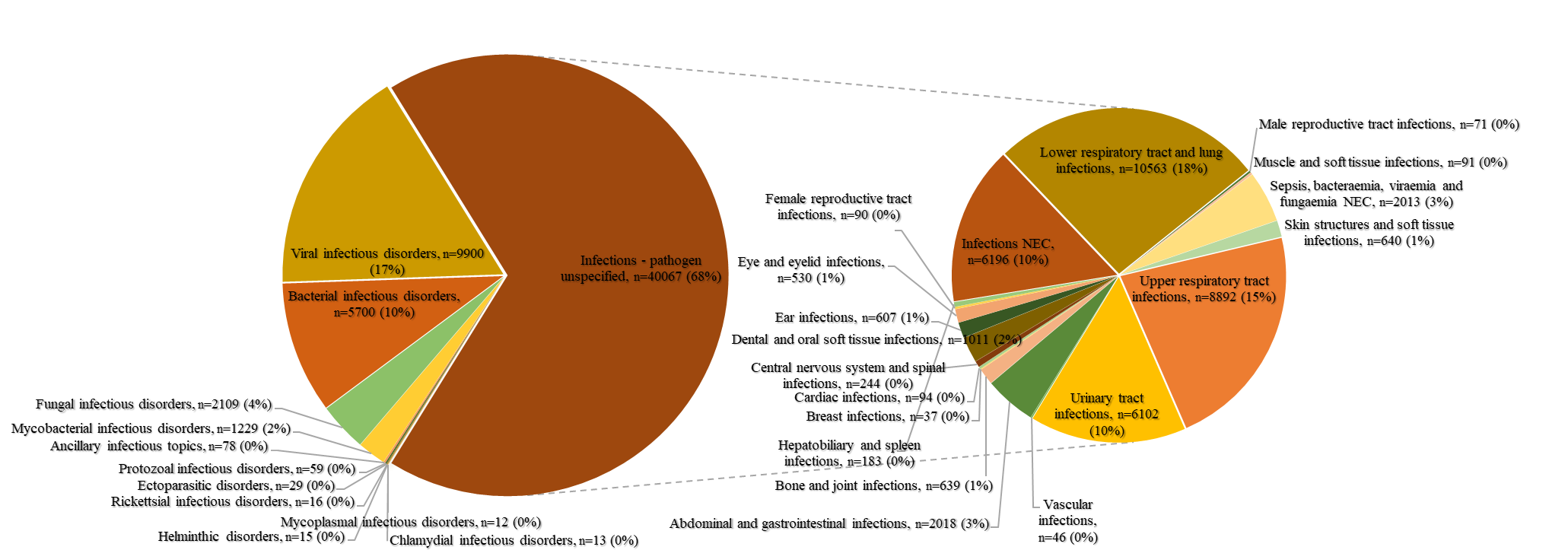


**Supplementary Figure 2.** The number of drugs for HLGT and HLT level

**
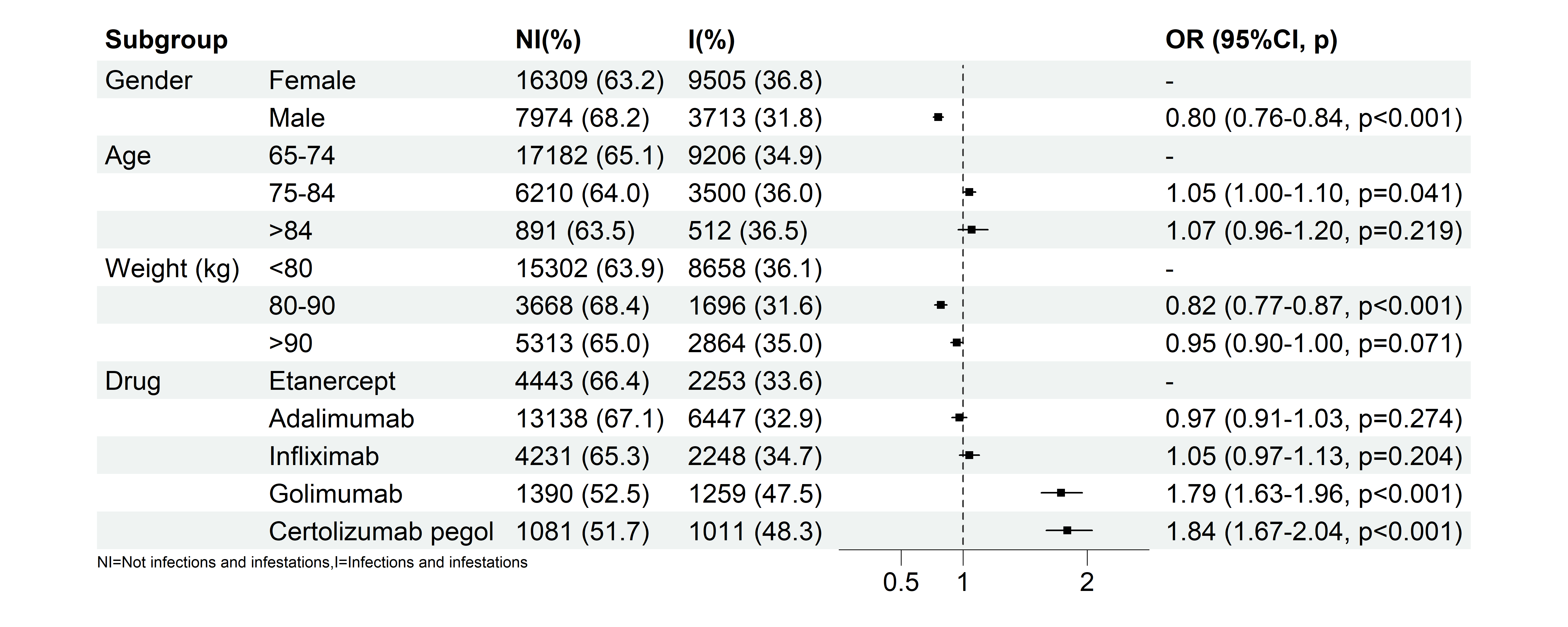
**

**Supplementary Figure 3.** Univariate logistic regression analysis of the odds ratio for adverse events of drug‑associated infections and infestations (NI=Not infections and infestations, I=Infections and infestations).
